# Genetic Predisposition to Neurological Complications in Patients with COVID-19

**DOI:** 10.3390/biom13010133

**Published:** 2023-01-09

**Authors:** Nikhil Shri Sahajpal, Alex R. Hastie, Maximilian Schieck, Ashis K. Mondal, Marc Felde, Caspar I. van der Made, Janet S. Chou, Adrienne G. Randolph, Thomas Illig, Michael C. Zody, Catherine A. Brownstein, Alan H. Beggs, Alexander Hoischen, Alka Chaubey, Ravindra Kolhe, on behalf of the COVID19hostgenomesv Consortium

**Affiliations:** 1 Department of Pathology, Medical College of Georgia, Augusta University, Augusta, GA 30912, USA; 2 Greenwood Genetic Center, Greenwood, SC 29646, USA; 3 Bionano Genomics, Inc., San Diego, CA 92121, USA; 4 Hannover Unified Biobank, Hannover Medical School, 30625 Hannover, Germany; 5 RESIST-Cluster of Excellence 2155, Hannover Medical School, 30625 Hannover, Germany; 6 Department of Human Genetics, Hannover Medical School, 30625 Hannover, Germany; 7 Department of Human Genetics, Radboud University Medical Center for Infectious Diseases (RCI), 6525 Nijmegen, The Netherlands; 8 Department of Internal Medicine, Radboud Institute for Molecular Life Sciences, Radboud University Medical Center, 6525 Nijmegen, The Netherlands; 9 Radboud Expertise Center for Immunodeficiency and Autoinflammation, Radboud University Medical Center, 6525 Nijmegen, The Netherlands; 10 Division of Immunology, The Manton Center for Orphan Disease Research, Boston Children’s Hospital, Harvard Medical School, Boston, MA 02115, USA; 11 Department of Anesthesiology, Critical Care, and Pain Medicine, The Manton Center for Orphan Disease Research, Boston Children’s Hospital, Harvard Medical School, Boston, MA 02115, USA; 12 Departments of Anesthesia and Pediatrics, Harvard Medical School, Boston, MA 02115, USA; 13 New York Genome Center, New York, NY 10013, USA; 14 Division of Genetics and Genomics, The Manton Center for Orphan Disease Research, Boston Children’s Hospital, Harvard Medical School, Boston, MA 02115, USA

**Keywords:** optical genome mapping, COVID-19, neurological complications, rare SVs, genetic predisposition

## Abstract

Several studies have identified rare and common genetic variants associated with severe COVID-19, but no study has reported genetic determinants as predisposition factors for neurological complications. In this report, we identified rare/unique structural variants (SVs) implicated in neurological functions in two individuals with neurological manifestations of COVID-19. This report highlights the possible genetic link to the neurological symptoms with COVID-19 and calls for a collective effort to study these cohorts for a possible genetic linkage.

## 1. Introduction

Several clinical studies have reported atypical symptoms of COVID-19, specifically the neurological complications that develop and persist during or after the SARS-CoV-2 infection. Older age, Caucasian ethnicity, male sex, and pre-existing neurological disorders have all been identified as common risk factors for developing neurological signs or syndromes in COVID-19 [1]. Although several studies have investigated and identified rare and common genetic variants associated with severe COVID-19, limited studies have investigated genetic determinants as predisposition factors for neurological complications. Moreover, these studies have remained limited to targeted gene analysis while investigating the association with neurological complications [2,3]. We previously identified rare structural variants (SVs) as predisposition factors associated with severe COVID-19 using optical genome mapping (OGM) technology [4]. Herein, we extend our work to investigate large rare/unique SVs as predisposition factors to neurological complications in COVID-19. We present 2 cases from our cohort of 114 severely ill COVID-19 patients, highlighting two rare SVs detected after filtering against >300 healthy controls and 106 age, sex, ethnicity, body mass index (BMI), co-morbidities, and COVID-19 severity-matched controls (Supplementary File Data Analysis) as candidates that may explain the neurological manifestations in these patients. 

## 2. A Case of Meningitis after COVID-19 

### 2.1. Clinical Course

A 57-year-old male with a pre-medical history of hypertension presented to the emergency room (ER) in December 2020 and reported an altered mental status observed at home. The patient was unable to follow commands and was agitated, restless, and combative, for which a level 2 code stroke was called and an urgent computed tomography (CT) for the head was performed on the patient. The CT was negative for any acute intracranial process, and the patient was admitted to the intensive care unit (ICU). The patient was uncooperative in the initial neurological examination but was observed to be awake and alert yet unable to follow commands, unable to frame complete sentences, and unable to walk without a deferred gait. The patient was reported to be positive for COVID-19 a month prior and that he had been feeling “under the weather” for the past few days. The initial concern on admission to the ICU was meningoencephalitis. A respiratory culture that was initially collected returned positive for *Pseudomonas* on day 4 and positive for methicillin-resistant *Staphylococcus aureus* (MRSA) on day 5. That day, a sepsis picture also developed, associated with hypotension and requiring norepinephrine. Eight days after admission, the patient began developing significant worsening of his renal function; therefore, continuous renal replacement therapy (CRRT) or intermittent hemodialysis (HD) was performed for the next two and one-half weeks, after which the patient had no dialysis or pressor requirements, leading to the removal of the HD line. At this time, the patient’s cerebrospinal fluid was also found to be negative for the SARS-CoV-2 virus and antibody. The patient also completed a 14-day course of meropenem for his *Pseudomonas pneumonia* infection. In the following neuro exam (day 30), the patient’s mental status was observed to be drowsy, but he was able to follow verbal commands. The motor response showed spontaneous movement in all extremities, against gravity, while the gait could not be assessed. The patient was discharged to a long-term acute care hospital with an ongoing altered mental state and meningitis one month after his initial presentation, and his case was lost for follow-up. 

### 2.2. Genetic Studies

**Figure 1:**
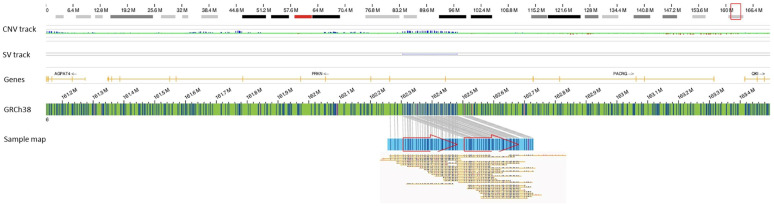
A ~176.7 kb duplication of chr 6 (chr6:162887379–162481424) disrupting the *PRKN* gene in one patient (case 53). The red arrows indicate a tandem duplication.

The patient’s genome was mapped retrospectively [using Bionano Genomics Saphyr® platform following the manufacturer’s protocols (Bionano Genomics Inc., San Diego, CA, USA)]. Genome analysis was performed using the de novo assembly pipeline included in the Bionano Access (v.1.6)/Bionano Solve (v.3.6) software, and two rare/unique SVs were identified in the *PRKN* and *CACNA2D1* genes. Most interestingly, a ~176 kb tandem duplication of chr 6 was identified in this patient, partially disrupting the *PRKN* gene and duplicating the coding exon 2 region of the gene (Figure 1). The exonic segmental copy number gain most likely results in an out-of-frame duplication, leading to truncation of the predicted protein (p.Arg41Valfs*98). The *PRKN* gene encodes a protein known as parkin, which is a component of a multiprotein E3 ubiquitin ligase complex that mediates the targeting of substrate proteins for proteasomal degradation and is also important for mitochondrial quality control by lysosome-dependent degradation of damaged mitochondria through mitophagy [5]. Homozygous or compound heterozygous mutation in the *PRKN* gene on chromosome 6q26 is associated with autosomal recessive juvenile Parkinson’s disease, type 2 (OMIM; 600116). Although few studies have argued that heterozygous variants in the *PRKN* gene do not cause Parkinson’s [6], several studies have shown that heterozygous carriers may develop late-onset Parkinson’s disease compared to compound heterozygous and homozygous carriers who had a significantly younger age at onset [7,8,9,10,11]. Interestingly, deletion or duplication of an entire exon (heterozygous or homozygous) has been reported to cause Parkinson’s disease [12]. 

**Figure 2:**
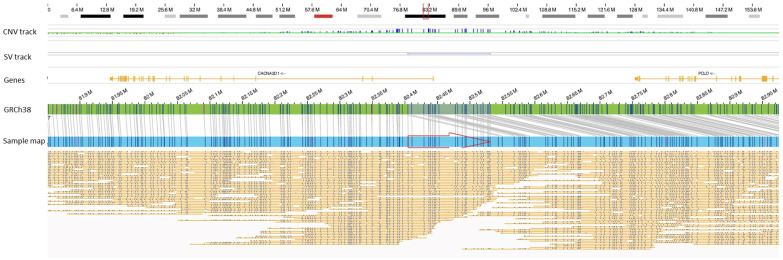
A ~126.5 kb duplication of chr 7 (chr7:82404497–82531042) disrupting the *CACNA2D1* gene in one patient (case 53). The red arrow indicates a tandem duplication.

To date, three cases of parkinsonism have been reported after COVID-19 [13,14,15]. In the three reported cases, two men (aged 45 and 58 years) were hypertensive, while the female (aged 35 years) had no pre-existing co-morbidities. The acute onset in three cases occurred 10–32 days after the COVID-19 diagnosis. One patient (the 58-year-old male) developed akinetic rigid syndrome, including myoclonus and opsoclonus compatible with encephalopathy, while the other two patients had asymmetric akinetic-rigid features with tremors and mild respiratory distress. The functional nigrostriatal neuroimaging was abnormal in all three cases, which implicated impaired dopaminergic nigrostriatal, but a genetic cause for parkinsonism or its predisposition was not identified in any of the three cases (genetic testing not performed on the female). Several parallels can be drawn between our case and the previously reported cases. We suspect that the rare/unique SV in the *PRKN* gene may have predisposed this patient (case 53) to Parkinson-like symptoms, but because of the continued secondary infection and associated pneumonia, the patient was never assumed to develop Parkinson’s and was not tested with functional nigrostriatal neuroimaging to rule in/out Parkinson’s. The acute onset and continued altered mental status in this patient after the SARS-CoV-2 infection implicates COVID-19 as a co-incidental trigger to elicit these neurological symptoms. Further, the ~126 kb duplication of chr 7 disrupts the *CCNA2D1* gene that encodes the α2δ subunit of the voltage-gated calcium channels (Figure 2). These channels have an important role in neurotransmission, and genetic aberrations in the gene have been associated with epilepsy and neuropsychiatric disorders [16]. Thus, we propose that the SVs in these two genes may be predisposition factors to neurological complications observed in this patient.

## 3. A Case of Encephalopathy

**Figure 3:**
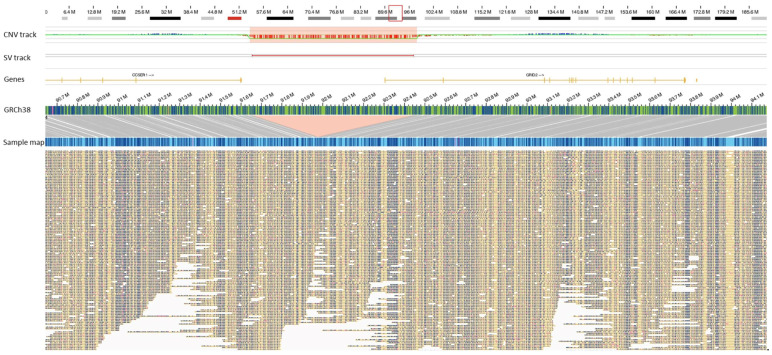
A ~784.3 kb deletion of chr 4 (chr4:91654881–92444482) disrupting the *GRID2* gene in one patient (case 23). The deletion is highlighted with the region in red.

A 71-year-old male, with a pre-medical history of diabetes mellitus and hypertension, was admitted to the ICU following a SARS-CoV-2 infection. The individual was diagnosed with ARDS, acute kidney failure, and encephalopathy; he was intubated for 13 days and managed with Azithromycin, Plaquenil, and Tocilizumab. The individual was discharged after 17 days in the ICU. A rare/unique SV, ~784 kb deletion of chr 4, that disrupted the *GRID2* gene (deleting 5′UTR and exon 1) was identified in this patient (Figure 3). The protein encoded by this gene is a member of the family of ionotropic glutamate receptors that are predominantly excitatory neurotransmitter receptors, expressed selectively in the cerebellar Purkinje cells [17]. Homozygous and compound heterozygous partial deletions of the *GRID2* gene (exons 4 and 2) have been identified to cause nystagmus, hypotonia with developmental delay in gross motor skills, encephalopathy with cerebellar ataxia, oculomotor apraxia, and pyramidal tract involvement [18]. Importantly, a 276 kb homozygous deletion of chr 4, partially deleting the *GRID2* gene and including the exon 1, has been associated with spastic paraplegia [19]. In addition, single nucleotide variants in the *GRID2* gene have been reported to cause congenital to mild adult-onset cerebellar ataxia [20]. Notably, anti-autoantibodies against the glutamate receptor delta 2 have been identified in patients with acute cerebellar ataxia and cerebellitis following respiratory tract viral infections, with improvement in symptoms once the infection is resolved [21,22]. Considering the evidence reported in the literature, the critical neurological role of the *GRID2* gene and the rare heterozygous deletion of exon 1 may have predisposed this patient to the neurological manifestations upon an acute SARS-CoV-2 infection.

## 4. Discussion

The neurological complications in COVID-19 patients are of significant concern and highlights our incomprehension of the post-infection sequela in these patients. Recently, mounting evidence demonstrates that a high prevalence of neurological complications is associated with morbidity/mortality in COVID-19 patients [1,23,24]. The neurological manifestations range from self-reported symptoms that include headache, anosmia, and ageusia as well as complications diagnosed with clinical evaluations that include acute encephalopathy, stroke, coma, seizure, status epilepticus, dysautonomia, meningitis, encephalitis, myelopathy, plegia, paralysis, aphasia, movement abnormalities, abnormal tone, abnormal brainstem reflexes, and sensory abnormalities [1]. A joint report from the GCS-NeuroCOVID Consortium and the ENERGY Consortium, which included 3743 patients from 28 centers across 13 countries, found neurological manifestations (self-reported neurological symptoms and/or clinically captured neurological signs and/or syndromes) in 82% of COVID-19 patients. The study identified older age, male sex, white race, and pre-existing neurological disorders to be associated with a higher risk of developing neurological signs or syndromes with COVID-19. Interestingly, self-reported neurological symptoms were associated with a reduced risk of in-hospital death, while clinically diagnosed neurological complications were associated with an increased risk of death [1]. 

These observational clinical studies have identified common risk factors associated with neurological manifestations in COVID-19 patients, but the mechanisms by which SARS-CoV-2 affects the brain remains unclear. Several hypotheses have surfaced: SARS-CoV-2 accesses the brain via olfactory mucosa and directly infects astrocytes [25], impairs blood flow to the brain tissue by infecting and impairing pericytes [26,27], or induces a deranged systemic immune response [28,29,30]. Additionally, inter-individual variability, evident from the neuro images and post-mortem brain experimental results exploring these hypotheses, further complicates and precludes the identification of a definitive mechanism [31,32,33,34]. Importantly, these studies highlight the inter-individual variability of the neurological manifestation in COVID-19 patients, which remains poorly understood. Given that several common and rare genetic factors have been identified that explain the predisposition to severe COVID-19, it seems plausible that a subset of patients with neurological complications may be genetically predisposed. To our knowledge, this present study is the first to report genetic determinants as predisposition factors to neurological complications in COVID-19 patients. 

We suspect that in the case of the 57-year-old male admitted due to meningitis, the harbored ~176 kb tandem duplication of chr 6 (the coding exon 2 of the *PRKN* gene) may have predisposed this patient to Parkinson-like symptoms. The acute onset and continued altered mental status in this patient after the SARS-CoV-2 infection implicates COVID-19 as a co-incidental trigger to elicit these neurological symptoms. Given that the genetic testing performed in the three previously reported cases remained limited to small sequence variation analysis [13,14,15], it would be interesting to see if those patients harbored any structural variations as observed in our study. Similarly, in the case of the 71-year-old male with the partial deletion of the *GRID2* gene, the incidental SARS-CoV-2 infection concomitantly resulted in the development of encephalopathy. Given that the ionotropic glutamate receptors are expressed selectively in the cerebellar Purkinje cells [17] and homozygous and compound heterozygous partial deletions of the *GRID2* gene (exons 4 and 2) have been identified to cause nystagmus, hypotonia with developmental delay in gross motor skills, encephalopathy with cerebellar ataxia, oculomotor apraxia, and pyramidal tract involvement, the SARS-CoV-2 infection appears to be a second hit that elicited the observed phenotypes.

## 5. Conclusions

This present study is an effort to identify candidate genes/SVs to understand the genetic predisposition to neurological manifestation in patients with COVID-19. This study identified SVs impacting genes implicated in neurological functions in patients with neurological manifestations of COVID-19, of which, two SVs in *PRKN* and *GRID2* genes are the most interesting candidates. The study highlights the possible genetic link to these neurological symptoms with COVID-19 and calls for a collective effort to study these cohorts for possible genetic linkage.

## Data Availability

All relevant data have been provided in the manuscript. Raw files for the optical genome mapping data will be available by request from the corresponding author and could not be uploaded to data-sharing domains because of IRB approval limitations.

## Supplementary Materials

The following are available online at https://www.mdpi.com/article/10.3390/biom13010133/s1, Data Analysis.
