# Supplementary material for: Genetic Predisposition to Neurological Complications in Patients with COVID-19
Source: Biomolecules. 2023 Jan 9;13(1):133. doi: 10.3390/biom13010133 (PMC9855758; doi:10.3390/biom13010133)
Supplement: Supplementary file 1 [file biomolecules-13-00133-s001.zip › biomolecules-2087901-supplementary.pdf]

Data Analysis

Rare/unique SVs were investigated to determine candidate gene/loci as potentially strong predisposing factors associated with neurological complications in patients with severe COVID-19. An unbiased whole genome analysis of SVs was performed to identify rare/unique genic SVs in these patients that did not appear in the population dataset (Bionano controls, comprising 267 individuals). The SVs in the neurological complication patients were further filtered by comparing age, sex, ethnicity, co-morbidities, and COVID-19 severity-matched controls (n=54). The rarity of the SV was confirmed using the public population control database (gnomAD). Additionally, only SVs disrupting the coding region(s) of the gene(s) were selected and reviewed for relevance with the phenotype. The Bionano control dataset includes 267 healthy individuals, of which 45 were African, 16 were admixed American, 17 were East Asian, 44 were European, 15 were South Asian, and 180 were of unknown origin.
